# Physiological and molecular mechanism of ginger (*Zingiber officinale* Roscoe) seedling response to salt stress

**DOI:** 10.3389/fpls.2023.1073434

**Published:** 2023-03-17

**Authors:** Miaohong Liu, Yao Lv, Bili Cao, Zijing Chen, Kun Xu

**Affiliations:** ^1^ College of Horticulture Science and Engineering, Shandong Agricultural University, Tai’an, China; ^2^ Collaborative Innovation Center of Fruit & Vegetable Quality and Efficient Production in Shandong, Tai’an, China; ^3^ Key Laboratory of Biology and Genetic Improvement of Horticultural Crops in Huanghuai Region, Ministry of Agriculture and Rural Affairs, Tai’an, China

**Keywords:** Ginger- *Zingiber officinale*, salt stress, osmotic regulation, antioxidant system, RNA-sequencing

## Abstract

We used ‘Shannong No.1’ experimental material to simulate higher salt concentration in ginger and analyzed the physiological responses of different parts of ginger seedlings under salt stress. The results showed that salt stress led to a significant decrease in fresh and dry weight of ginger, lipid membrane peroxidation, increased sodium ion content and enhanced activity of antioxidant enzymes. Compared with the control, the overall plant dry weight of ginger under salt stress decreased by about 60%, and the MDA content in roots, stems, leaves, and rhizomes increased by 372.27%, 184.88%, 291.5%, and 171.13%, respectively, and the APX content increased by 188.85%, 165.56%, 195.38%, and 40.08%, respectively. After analysis of the physiological indicators, it was found that the roots and leaves of ginger were the most significantly changed parts. We analyzed the transcriptional differences between ginger roots and leaves by RNA-seq and found that they jointly initiated MAPK signaling pathways in response to salt stress. By combining physiological and molecular indicators, we elucidated the response of different tissues and parts of ginger to salt stress during the seedling stage.

## Introduction

Ginger (*Zingiber officinale Roscoe*) is a versatile vegetable crop with a savory flavor belonging to the ginger family. Ginger benefits from its beneficial properties such as pungent aroma and pharmacological activity and is used as a food, spice, supplement and flavoring as well as a traditional medicine (Kiyama, 2020). It is one of China’s key export-earning vegetables. Ginger has been used as a digestive and anti-inflammatory medicine by the Chinese for at least 2,500 years. Ginger is an important crop in Shandong Province, China (Liu et al., 2020). The rhizome, as the main product organ of ginger, is an underground organ along with the root and is influenced by the soil environment. Ginger has the characteristics of continuous cultivation, which, together with the large number of fertilizers applied by farmers during the cultivation process, can lead to secondary salinization in the main ginger-producing areas, which echoes the following situation.

Salinization of soil and freshwater resources by natural processes and human activities has become a growing problem affecting environmental services and socio-economic relations (Ondrasek et al., 2022). Due to the capillary action of groundwater, the soluble salt in the soil will accumulate on the surface with the increase of evaporation. High salt content will lead to different degrees of salinization (Feng et al., 2019). By 2050, salinization is anticipated to have varied effects on nearly half of agricultural land (Kumar et al., 2020). China’s arable land resources are scarce, less than 40% of the world average, and with a large population, the arable land per capita is only 0.092 hectares (Zhang and Wang, 2021). By analyzing the soil in the main ginger-producing area of Shandong province, we found that the average salt content ranged from 0.22% to 0.36%, and the highest salt content was up to 0.5%. This has a noteworthy effect on the number of gingers grown and the cost-effectiveness of land utilization. There are now two primary techniques for reducing soil salinity: trying to add chemicals and generating salt-tolerant plant types *via* biotechnology. In contrast, the former can lead to secondary salinization and is expensive. Therefore, it is very important to understand the mechanism of plant salt tolerance by cultivating salt-tolerant plants (Hao et al., 2021).

Some scholars have discovered *via* thorough comparison studies that the relative importance of fresh weight and electrolyte leakage is the clear and concise marker to evaluate the salt stress tolerance of Chinese cabbage (Li et al., 2021). Salt-induced osmotic stress in tomato exhibited lower relative water content, where higher Na/K ratios indicated ionic toxicity (Zhou et al., 2022). Malondialdehyde, endogenous proline and electrolyte leakage, antioxidant defense systems, and sodium ion content were all found to increase significantly after NaCl stress (Sofy et al., 2020). Salt stress caused an increase in antioxidant enzyme activities such as ascorbate peroxidase (APX), peroxidase (POD), catalase (CAT), superoxide dismutase (SOD) and proline content in Aloe vera compared to the control (Kavian et al., 2022). Several DEGs enriched in plant signal transduction pathways were highly expressed in oilseed rape seedlings under salt stress (Wang et al., 2022b). Therefore, in this study, we selected the ginger variety ‘Shannong No.1’ as the experimental material to study the changes in the overall osmoregulatory system and reactive oxygen metabolism of ginger during the more sensitive seedling stage (80 d). The possible genes and pathways connected to the stress-induced response were explored in this work, which employed RNA sequencing to investigate changes in the expression of several genes in ginger seedlings under salt stress. The breeding of salt-tolerant cultivars and the appropriate use of land resources both benefit tremendously from this work.

## Materials and methods

### Plant material and experimental design

The experiment was conducted at the experimental station of Shandong Agricultural University located in Tai’an, eastern China (36°09′ N, 117°09′ E). NaCl and Na_2_SO_4_ (1:1) mass ratio was used as the salt stress to simulate neutral salt stress of 0.5% in the primary ginger-producing region. The substrate was poured into special ginger cultivation bags after the neutral soil, which weighed 10 kg and had been sieved to remove impurities and stones, had been evenly mixed with 25 g of sodium chloride and 25 g of sodium sulfate (produced by Tianjin Kai tong Chemical Reagent Co., Ltd., China). The variety of ginger was chosen as ‘Shannong No.1’. Ginger sprouts to a uniform size of about 1 cm, leaving a healthy bud to be cultured in a culture bag in mid to early May. The CK treatment was the same as above, without salt added. Samples were taken at the ginger seedling stage (80 days of cultivation) and relevant indicators were measured.

### Measuring methods

#### Measuring parts

Contains root, stem, leaf, and rhizome. The roots of ginger include fibrous and fleshy roots. The fibrous roots are the main absorbing organs of ginger, while the fleshy roots mainly serve to support the plant upright and store nutrients. The underground stem of ginger is known as the rhizome. The rhizome is the underground rhizomatous fleshy rhizome that forms when the base of the ginger expands and serves a reproductive function and is the main product or food organ of ginger where most of the nutrients of ginger are stored.

#### Water content

Plants of the same size were selected for sampling in each treatment. Weigh the fresh weight of each part, then record the dry weight by drying. Total water content The determination of total water content was slightly modified according to Meher et al(Shivakrishna et al., 2018). Free water and bound water Specific measurement method reference Vijaya et al. (Singh et al., 2006).

#### Osmotic regulation system

Five identically sized and positioned leaves from a ginger seedling were chosen for each treatment. The conductometer Leici-ddb-303a was used. Li et al. Are referenced in detail (Li et al., 2021). Proline content is determined by the NINHYDRIN method with specific reference to Chen et al. (Chen et al., 2019). The tissue of ginger seedlings was washed, dried, and digested with H_2_SO_4_-H_2_O_2_. The solution was analyzed by emission spectrometer.

#### Antioxidant system

After sampling at the ginger seedling stage, the samples were quickly washed and dried, and the supernatant was ground to extract as the enzyme source (Gong et al., 2014). To identify the buildup of superoxide anion, we utilized nitro blue tetrazolium (Xia et al., 2009). Using Patterson et al. Approach, the amount of H_2_O_2_ was evaluated (Patterson et al., 1984). The barbituric acid technique was used to measure the amount of malondialdehyde (Wada et al., 2011). Using the extraction enzyme solution technique, the activities of Superoxide dismutase, Peroxidase, and Catalase were assessed (Roldán et al., 2008; Chapman et al., 2019). APX activity was determined using information from Nakano et al. Research (Nakano and Asada, 1981).

#### Transcriptome analysis

Transcriptome sequencing was performed at Beijing Novo Gene Company (Beijing, China). We get the reference genome and gene model annotation file from NCBI. In Supplementary **Table 1**, the primers for the chosen DEGs genes’ RT-qPCR are given. It was determined to use Zo RPII as the internal reference gene. (Lv et al., 2020). qPCR was performed using Hi Script III RT Super Mix (Vazyme, China). See the instructions for the specific steps. The relative representation algorithm is detailed in the references (Li et al., 2021).

### Statistical analysis

All plant samples used in this investigation were drawn at random. GraphPad Prism 6.0, Excel 2016, and the DPS package were used to process, plot, and statistically analyze the data (DPS for Windows, 2009), testing for variations between treatments using Duncan’s new multiple range tests; the significance threshold was P ≤0.05.

## Results

### Fresh weight, dry weight, growth

**Figure 1:**
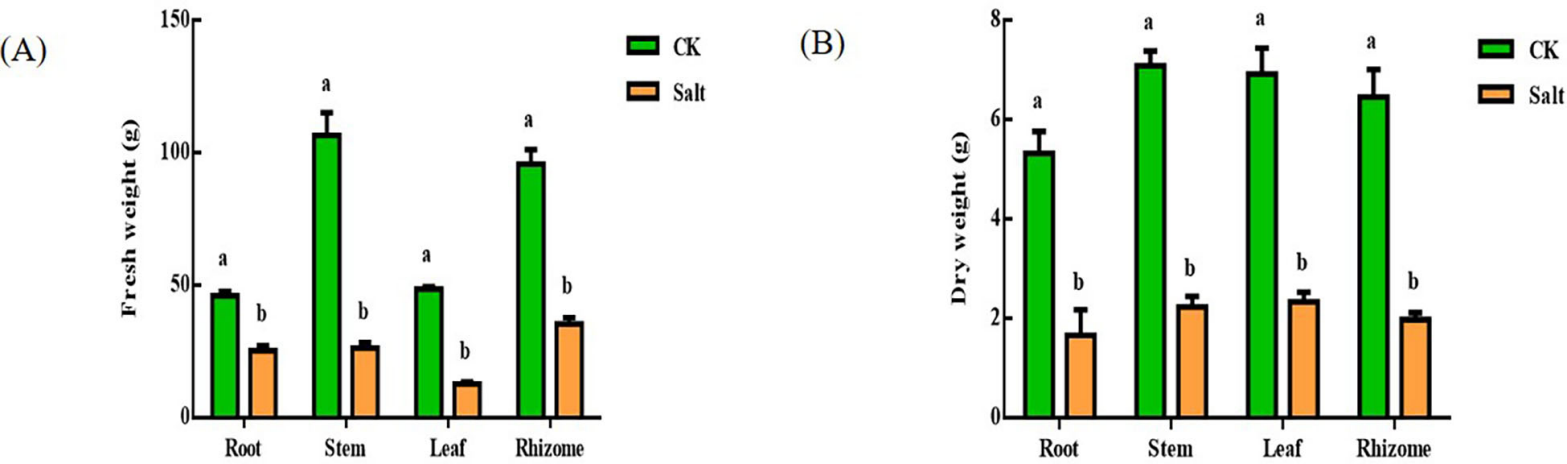
Effect of salt stress on the water content of various parts of ginger seedlings. **(A)** Fresh weight. **(B)** Dry weight. The lowercase letters a and b mean the letter markers for significant difference analysis.

Salt stress reduced the development of sensitive seedlings, as seen in **Figure 1**. Root, stem, leaf, and rhizome fresh weights all dropped by 44.96%, 75.34%, 73.81%, and 63.03%, respectively. The difference between rhizome and leaf was the most obvious. Root, stem, leaf, and rhizome fresh weights all fell by 68.69%, 68.47%, 66.2%, and 69.25%, respectively. The whole plant’s dry weight dropped by around 60%.

### Leaf water content, electrical conductivity, free and bound water

**Figure 2:**
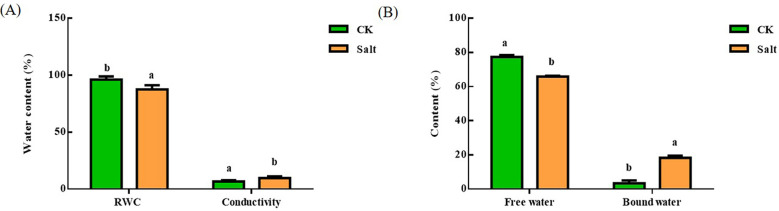
Effect of salt stress on the relative water content, conductivity and free water bound water content of ginger seedling leaves. **(A)** Relative water content and electrical conductivity. **(B)** Free and bound water. The lowercase letters a and b mean the letter markers for significant difference analysis.

Under salt stress, **Figure 2** depicted how the water content of ginger leaves varied. The relative water content dropped by 9.04% in comparison to the control group, but electrical conductivity rose by roughly 40%. The amount of free water in leaves reduced by 14.78% when compared to the control, but the amount of irreducible water rose by about four times.

### Proline, malondialdehyde, superoxide anion production rate, and hydrogen peroxide content

**Figure 3:**
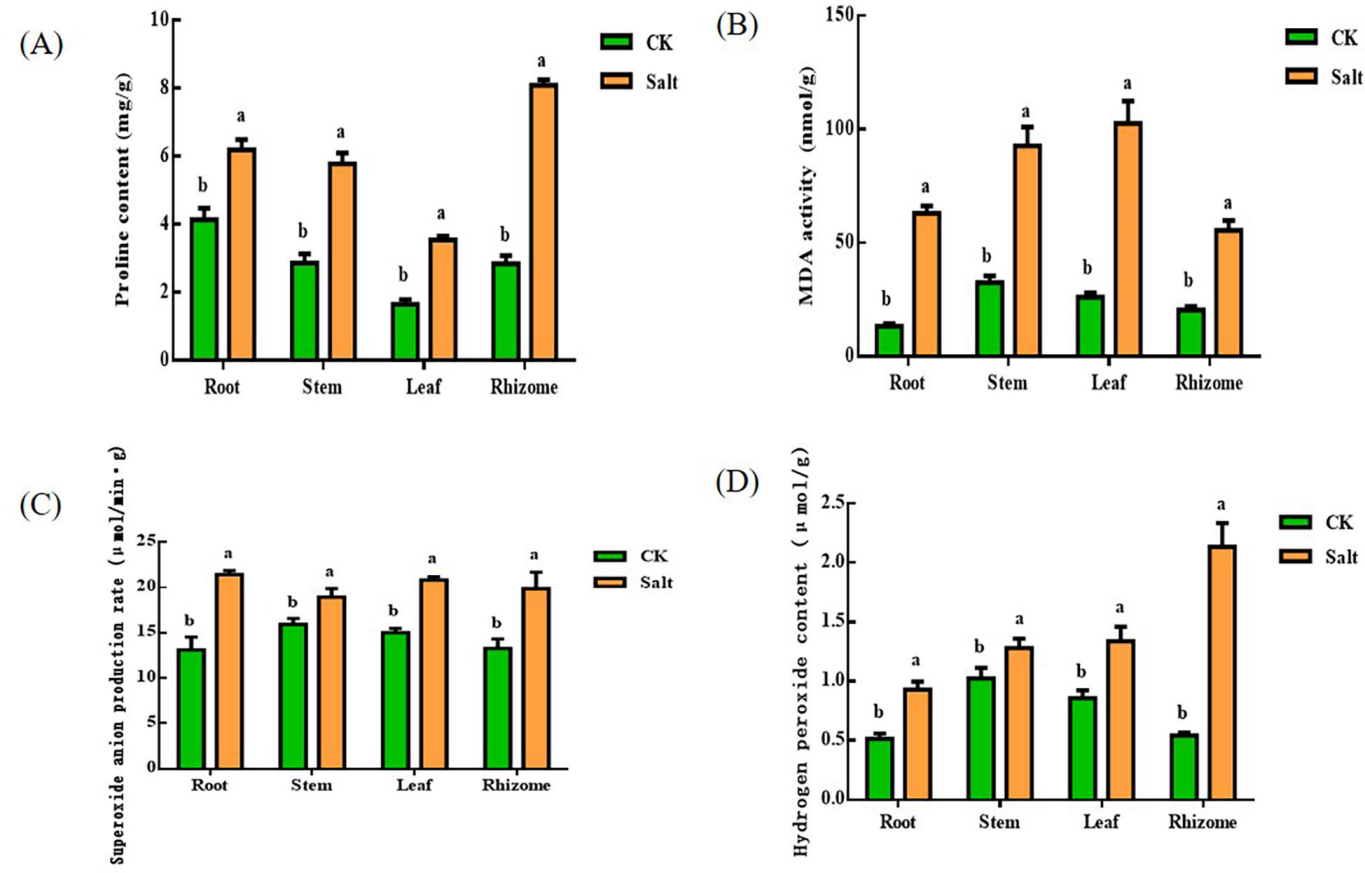
Effects of salt stress on proline, malondialdehyde, superoxide anion production rate and hydrogen peroxide content in different parts of ginger seedlings. **(A)** The proline content. **(B)** The malondialdehyde content. **(C)** The rate of superoxide anion production. **(D)** The hydrogen peroxide content. The lowercase letters a and b mean the letter markers for significant difference analysis.

**Figure 3** demonstrated that, during the seedling stage, ginger tissues had higher proline and malondialdehyde concentrations due to salt stress. The proline content in roots, stems, leaves, and rhizomes grew by 49.96%, 101.56%, 113.85%, and 184.3%, respectively, when compared to the control group. The most notable impact of salt stress was on the root and rhizome. The levels of MDA in various tissues elevated by 372.27%, 184.88%, 291.5%, and 171.13%, respectively, in comparison to the control group. The most obvious part of the change is the root.

In all tissues of ginger seedlings under salt stress, superoxide anion production and hydrogen peroxide levels increased. Superoxide anion concentrations rose by 63.36%, 18.99%, 38.78%, and 49.52%, respectively, in comparison to the control. Among them, the change of root system was more significant. In addition, the superoxide production rate of tissue increased by 81.39%, 25.03%, 56.19%, and 292.75%, respectively. The most significant changes occurred in the rhizome.

### Superoxide dismutase, peroxidase, catalase, ascorbate peroxidase content

**Figure 4:**
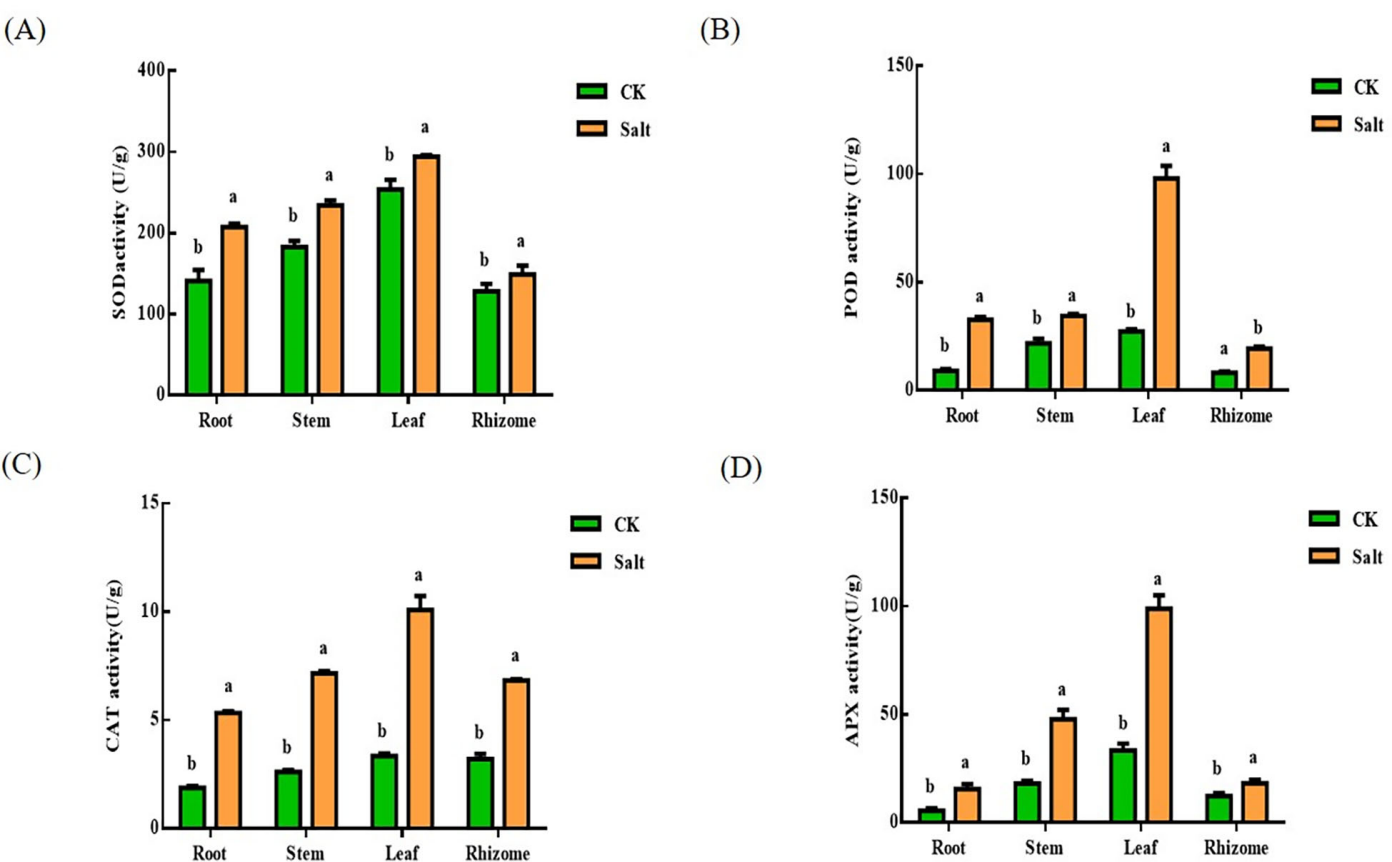
Effects of salt stress on SOD, POD, CAT and APX contents in various parts of ginger seedlings. **(A)** The SOD content. **(B)** The POD content. **(C)** The CAT content. **(D)** The APX content. The lowercase letters a and b mean the letter markers for significant difference analysis.

It can be understood by **Figure 4**. Under salt stress, SOD, POD, CAT, and APX concentrations rose. SOD levels rose by 46.95%, 27.9%, 15.88%, and 16.11%, respectively, in relation to the control. The most dramatic changes take place at the root. The POD content in different tissues increased by 261.28%, 57.79%, 258.47% and 136.6% respectively. The most significant changes are in the root and leaf sections. The content of CAT increased by 183.75%, 172.98%, 201.12% and 113.13% respectively. Like the POD, the most obvious changes are in the root and leave. The content of APX increased by 188.85%, 165.56%, 195.38% and 40.08% respectively. The changing trend was consistent with that of POD and CAT, and the changes in root and leaf were the most obvious.

### Sodium and potassium content

**Figure 5:**
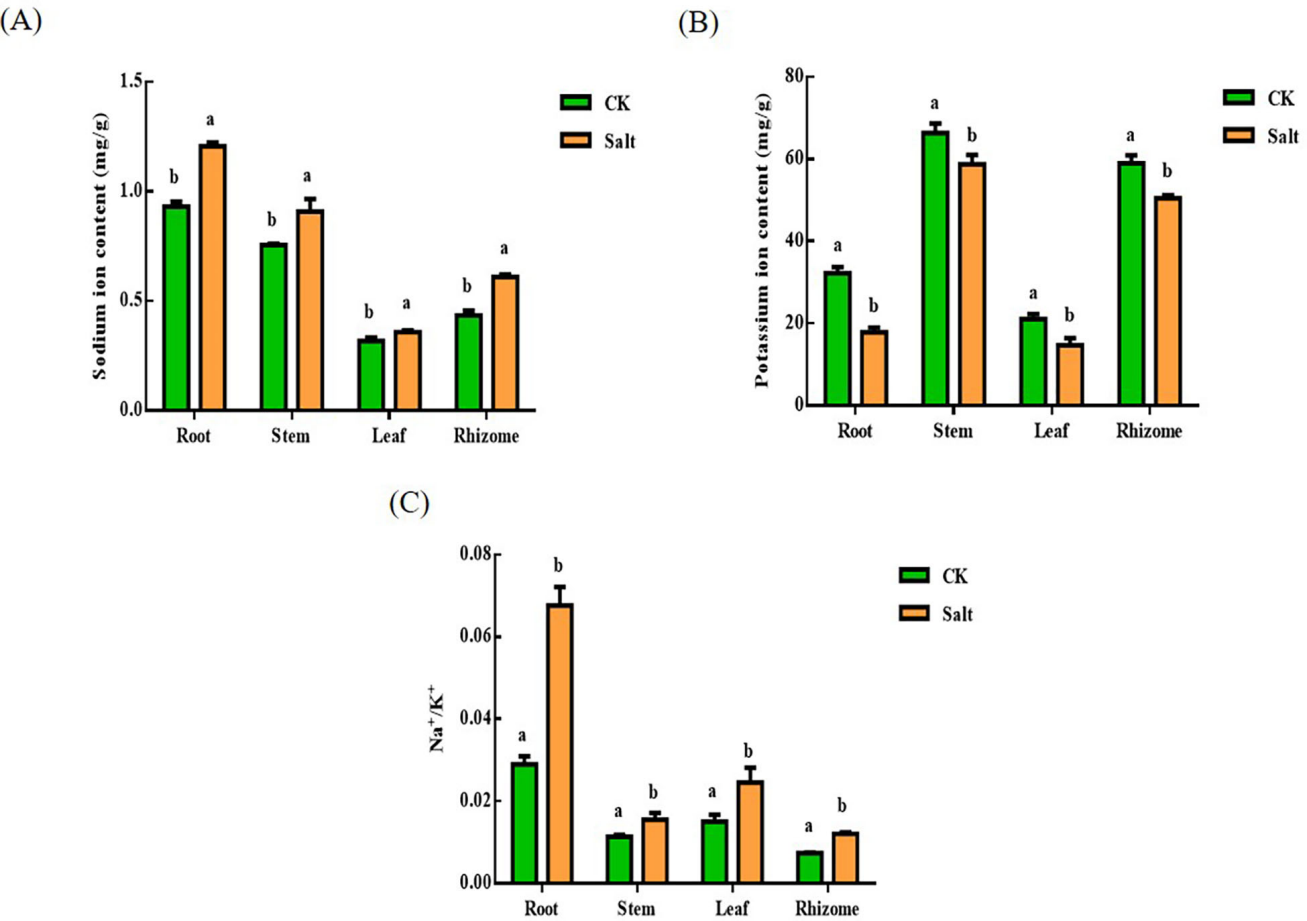
Effect of salt stress on the ion content of each part of ginger seedlings. **(A)** The sodium ion content. **(B)** The potassium ion content. **(C)** The ratio of sodium ion content to potassium ion content. The lowercase letters a and b mean the letter markers for significant difference analysis.

Salt stress resulted in a reduction in potassium ion content and an increase in sodium ion content, as seen in **Figure 5**. There in ginger root, stem, leaf, and rhizome, respectively, the sodium ion level rose by 29.53%, 20.3%, 12.49%, and 40.7% when compared to the control. The more prominent parts are the root and rhizome. And the content of potassium in each tissue decreased by 44.2%, 11.28%, 27.91%, and 15.91%, respectively. The most prominent part is the root. The ratio of sodium ions to potassium ions similarly exhibited an increasing trend after salt stress.

### Transcriptome sequencing and RT-qPCR validation

**Figure 6:**
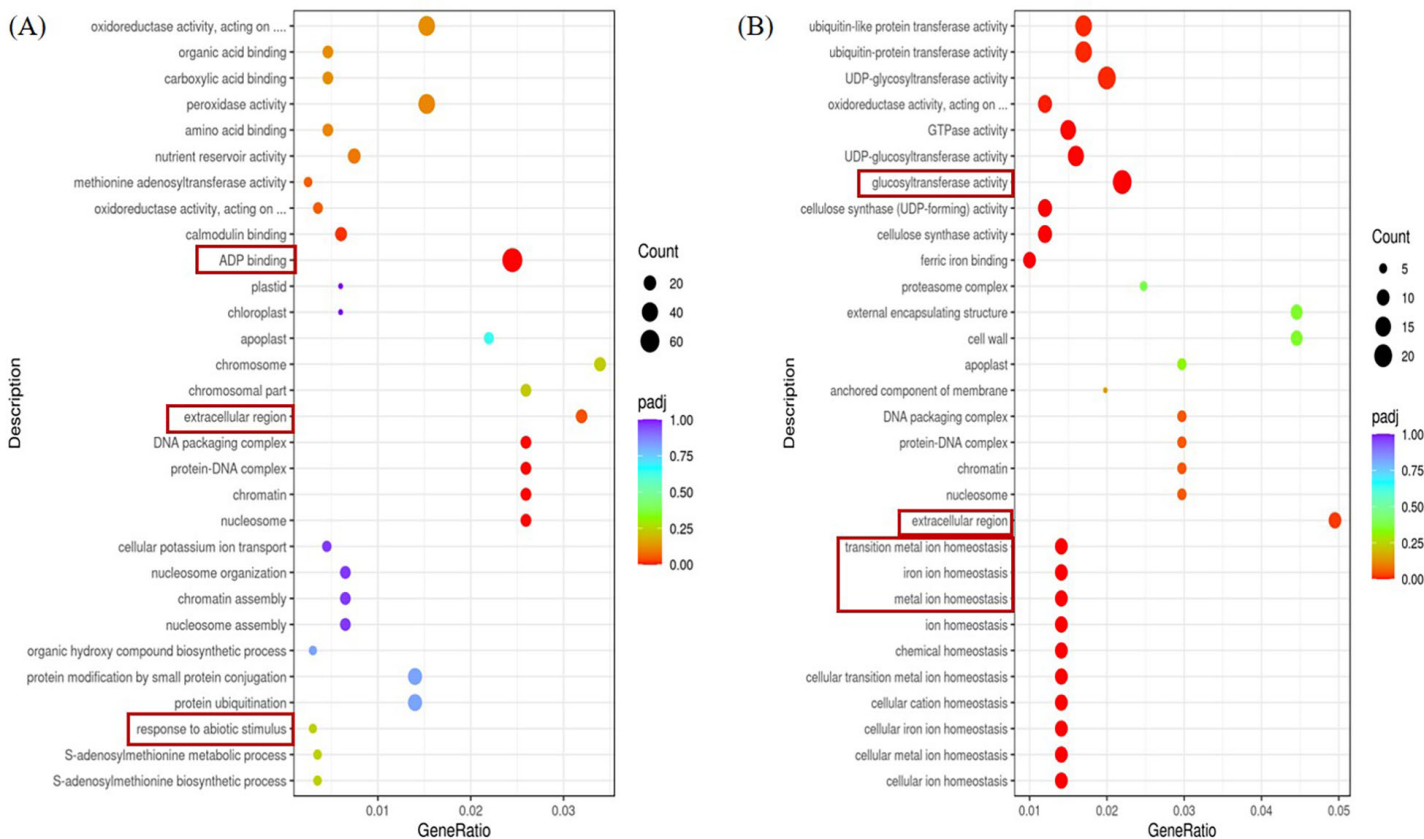
GO terms for the top 20 significant DEGs. **(A)** The first 20 GO terms in the root parts were significantly enriched at the P<0.05 level in the comparison between CK and salt stress treatments. **(B)** The first 20 GO terms in the leaf parts were significantly enriched at the P<0.05 level in the comparison between CK and salt stress treatments.

Scatterplot **Figure 6** is the top 30 terms that make the most sense. The left A panel shows the GO functional enrichment analysis of differential genes between the stress group and the control group in ginger seedling roots. Differential genes are primarily concentrated in the ADP-binding area in biological processes, whereas they are primarily concentrated in the extracellular region in cellular fractions. In Molecular Function, response to abiotic stimulus was enriched with more differential genes. The differential genes in the biological process, cellular component, and molecular function were significantly enriched in glucosyltransferase activity, as shown in the right B panel, which also shows the enrichment analysis of the heterozygous GO function in the stress group and the control group in the leaf parts of ginger seedlings. In contrast to the control, the extracellular portion of the cellular component in both roots and leaves under salt stress was enriched in the differential genes.

**Figure 7:**
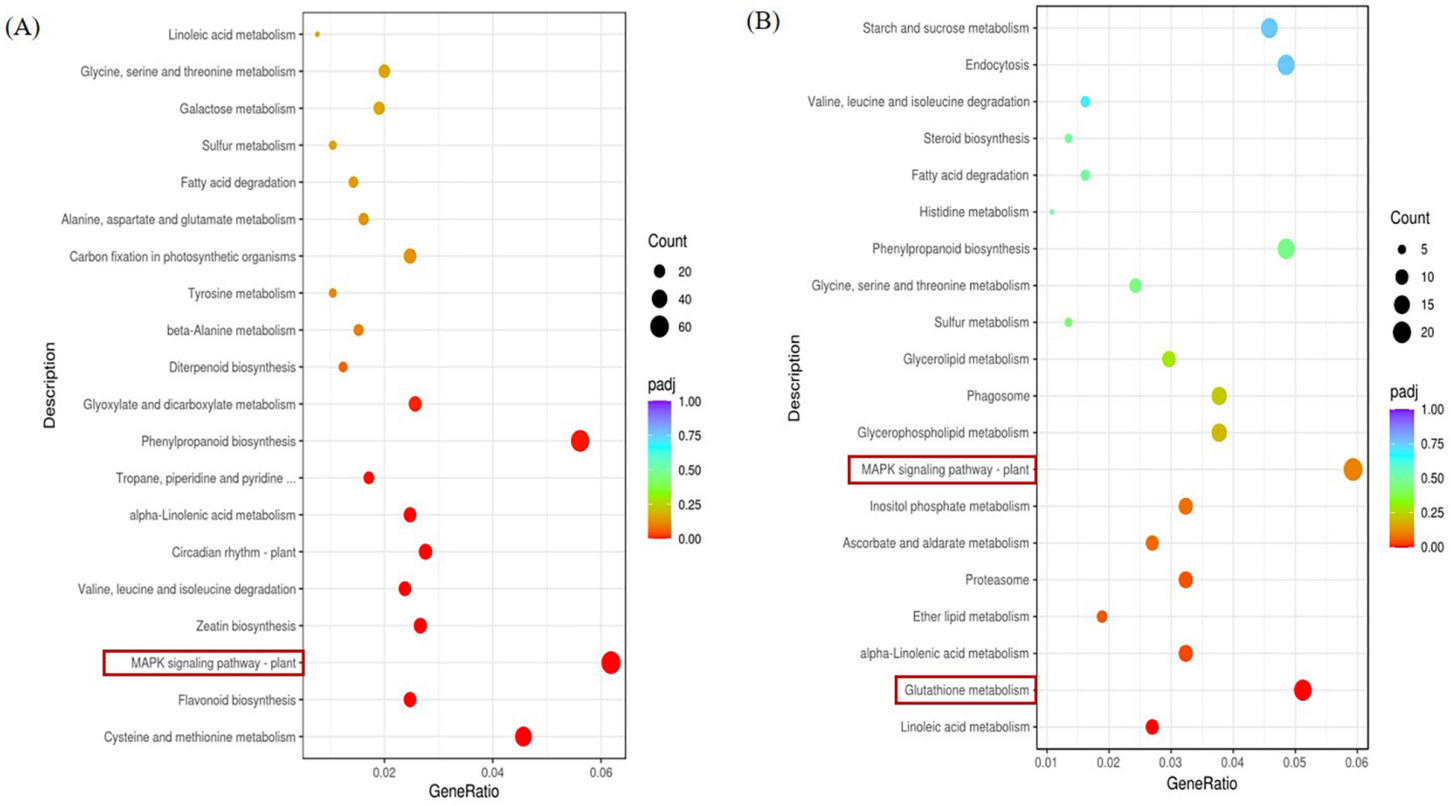
KEGG pathway of DEGs KEGG. **(A)** KEGG pathway significantly enriched by DEGs in the root parts compared with salt stress treatment in CK, P < 0.05. **(B)** KEGG pathway significantly enriched by DEGs in leaf parts compared with salt stress treatment in CK, P < 0.05.

We can be informed by **Figure 7**. The left KEGG pathway enrichment analysis of the stress group and the control group in the ginger seedling roots is shown in a panel. The right B panel displays the enrichment analysis of the KEGG pathway in the stress group and control group in the leaf of ginger seedlings, with MAPK signaling pathway - plant being the more important pathway. In comparison to the control, the roots and leaves of the salt-stressed plant had more abundant of KEGG pathway associated with the MAPK signaling pathway. The plant has a higher KEGG pathway enrichment.

**Figure 8:**
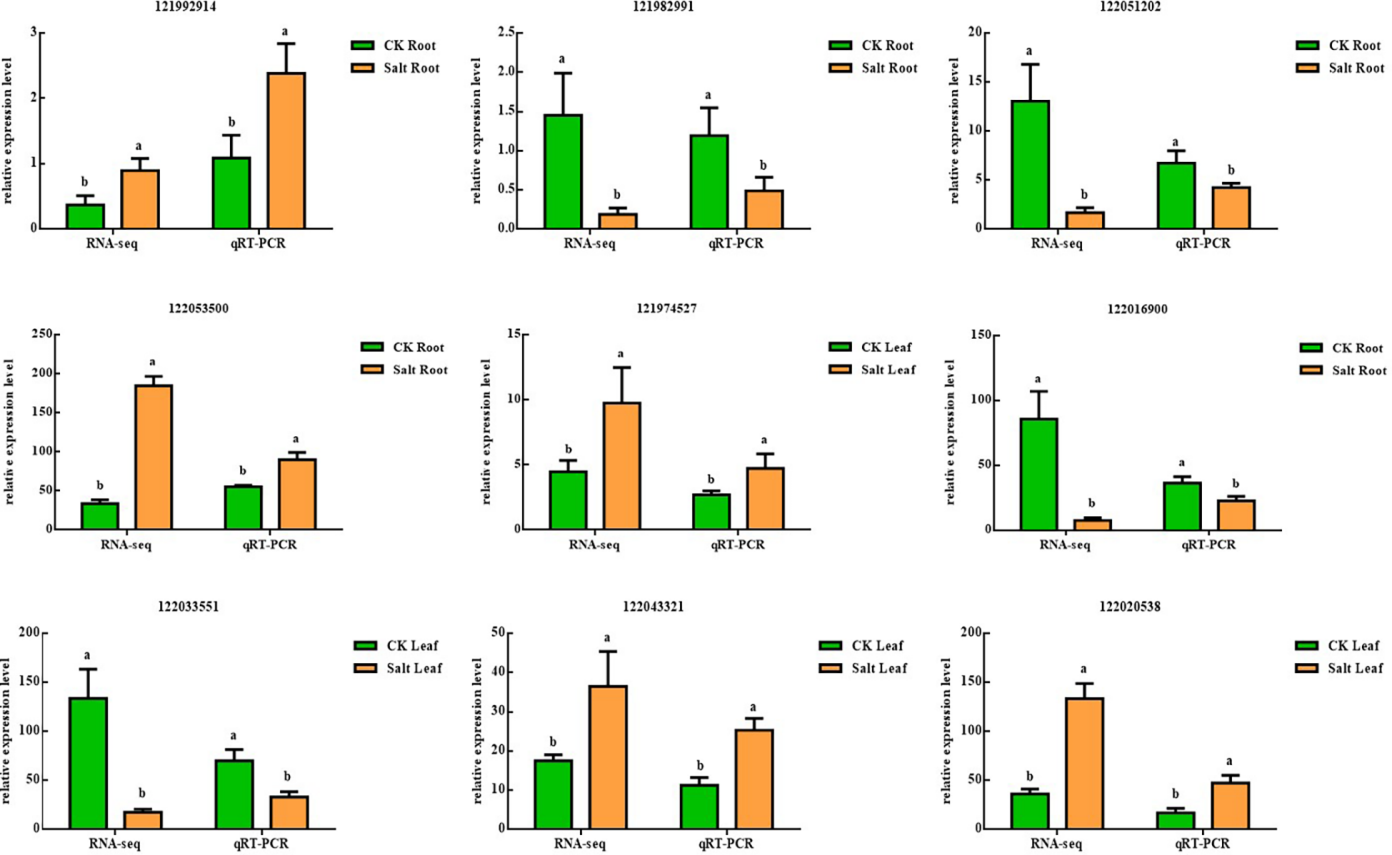
Validation of nine differentially expressed genes using real-time quantitative PCR in salt-stressed ginger seedling roots and leaves, P < 0.05. The lowercase letters a and b mean the letter markers for significant difference analysis.

**Figure 8** showed real-time fluorescent quantitative PCR results and RNA-seq results showing the same underlying trend, although expression ploidy is slightly different, indicating that transcriptome data are accurate and reliable.

## Discussion

Growth inhibition, rapid development, and aging are prominent signs of salt stress damage since prolonged exposure may cause plants to die (Acosta-Motos et al., 2017). Growth inhibition is a major impairment leading to other symptoms and may also lead to programmed cell death under severe salinity shocks (Jouyban, 2012). Tanveer et al. found that salt pressure forced tomato seeds to reduce germination time by 27.6% and salt tolerance by 27.6%. Seedling length and viability decreased by 24.33% (Tanveer et al., 2020). Researchers noted that the initial drop in development was brought on by the osmosis of salt outside the root, but the following reduction in growth was brought on by the failure to keep the salt from reaching dangerous levels in the carrying leaves (Geilfus, 2018). According to Mathias J, greater osmotic content may have contributed to the relative water content of pepper leaves being higher in salt-tolerant types than salt-sensitive ones (Hand et al., 2017). After salt stress, tomatoes experience lipid peroxidation salt stress, which Siddiqui hypothesized may be generated by the buildup of MDA in the membrane, which is driven by ROS (Siddiqui et al., 2017). Lipid peroxidation is a frequently used indicator of membrane damage brought on by stress (Sekmen et al., 2012). In this case, salt-tolerant varieties were able to reduce lipid peroxidation levels by reducing MDA content. Mahmoud et al. showed that similar to Lipid peroxidation, H_2_O_2_ content in the leaves of cultivars increased with varying degrees under salt stress (Akrami and Arzani, 2018). Interestingly, in our study, after salt stress treatment of ginger, the MDA content level of each part was positively correlated with H_2_O_2_ content and superoxide anion production rate, indicating that the level of lipid peroxidation was increased. It is worth noting that the proline content of each part also showed an upward trend to varying degrees.

Recent studies have shown that antioxidant enzyme activity is induced to increase under salt stress, suggesting that resistance to it is protective against stress responses (Cai et al., 2021). Susana discovered that silicon controlled the activities of antioxidant enzymes and nitrogen metabolism to mitigate the negative effects of salt on sunflower plants (Conceição et al., 2019). The findings indicated a positive association between SOD, CAT, and POX activity in tall fescue leaves as an increase in SOD activity was followed by increases in CAT and POX activity, which reduced the buildup of H_2_O_2_ caused by abiotic stress (Fu et al., 2015). Wang mentioned that melatonin’s impact on cucumber seedlings under salt stress is connected to the formation of hydrogen peroxide (Zhang et al., 2020). According to this research, salt stress dramatically enhanced the levels of SOD, POD, CAT, and APX in different tissues and sections of ginger during the seedling stage. Combined with the ROS products discussed and analyzed above, it is speculated that this phenomenon may be the stress response of ginger under salt stress. A potassium deficit should be noted as a potential cause of growth limitation. The most hazardous sodium-potassium ratio is high. In order to sustain enough potassium nutrition under salt stress, plants must thus use a highly selective and high-affinity potassium absorption mechanism (Jouyban and Sciences, 2012). It’s interesting how this matches the findings of this research. After salt stress, it was discovered that the Na^+^/K^+^ ratio dramatically rose, particularly in the ginger root. Kongake et al. found that Na^+^ content in salt-sensitive rice roots directly increased with increasing NaCl concentration, resulting in decreased plant water content and growth inhibition, presumably resulting in membrane damage due to Na^+^/K^+^ and Na^+^ toxicity (Siringam et al., 2011). Zeeshan found that barley and wheat varieties were more salt-tolerant by making the sodium-to-potassium ratio more balanced and by increasing ROS scavenging enzyme activity(Zeeshan et al., 2020).

DREB is a TFS that regulates the expression of various stress response genes in plants. In this study, the gene 121982991 in Ginger Root was closely related to the biological function of DREB after salt stress. Some studies have shown that the DREB gene BADBL1, derived from the desiccation-tolerant moss, makes transgenic Arabidopsis thaliana tolerant to osmotic pressure and salt stress. This may be accomplished by raising the activity of its antioxidant enzymes, controlling the expression of stress-related genes in plants, and modifying the lignin’s production (Liang et al., 2021). Additionally, it has been shown that the functional homolog GHDREB1 may enhance the freezing, salt, and osmotic tolerance of transgenic Arabidopsis thaliana (Lata and Prasad, 2011). The APETALA2/Ethylene Responsive Factor Transcription Factor family is one of the mechanisms mentioned by Sameer. This family regulates salt tolerance by binding to upstream the DNA of genes that function specifically in the salt-tolerant pathway. As a result, the DREB protein increased the expression of these salt-tolerant genes (Hassan et al., 2022). In addition, we discovered that the biological function of MYB under salt stress was tightly connected to the ginger root gene 122051202. Many of the MYB proteins’ downstream targets have been discovered, and these proteins help plants tolerate salt. IBMYB308, a sweet potato isozyme, was shown in Wang’s work to enhance transgenic tobacco’s salt tolerance (Wang et al., 2022a). According to Li’s work, Fvmyb82 from strawberries may be crucial for regulating downstream-related genes in Arabidopsis during salt and cold stress (Li et al., 2022). Through an ABA-dependent mechanism, it was discovered that the maize MYB transcription factor ZMMYB3R increases tolerance to salt and drought stress (Wu et al., 2019). The response to abiotic stressors is significantly influenced by the Mitogen-Activated Protein Kinase (MAPK) cascade. The biological activity of MAPK following salt stress was discovered to be strongly connected to the ginger leaf gene 122020538, according to this research. Ying et al. discovered that GHCIPK6A’s function in scavenging ROS and MAPK signaling pathways was responsible for the increased salt tolerance of transgenic upland cotton (Su et al., 2020). According to Hoang et al., the MAP kinase MPK6 is activated and interacts with MYB41, increasing salt tolerance in Arabidopsis thaliana. This suggests that MAP kinases, which function as signal sensors and transmit osmotic pressure signals to the appropriate effectors, aid plant cells in becoming acclimated to high salt concentrations (Hoang et al., 2012). By raising the expression of stress-related genes and the activity of antioxidant enzymes under salt stress, FTMAPK1 improved the stress resistance in Yao’s research (Yao et al., 2022). The grape-isolated protein VVMAPK9, when overexpressed in Arabidopsis thaliana, significantly improved the plant’s ability to withstand salt stress. In addition, overexpressed VVMAPK9 in grape Calli improved the Calli’s capacity to scavenge reactive oxygen species, which further improved the salt-resistant mechanism (Ji et al., 2022).

## Conclusions

Ginger uses the rootstock as its main edible organ, so the soil environment is particularly important and sensitive to its underground organs. Regardless of where ginger is grown globally or in China, soil salinity problems have a significant impact on plant growth and development. The results of this study showed that salt stress inhibited ginger growth with increased osmoregulatory substances, increased reactive oxygen species, increased sodium ion content, and enhanced antioxidant enzyme activity. Analysis of the indicators of each tissue part revealed that roots and leaves were the most significantly changed parts. After analyzing the transcriptional differences between roots and leaves by RNA-seq, it was found that they jointly initiated MAPK signaling pathway in response to salt stress. This work effectively identifies the physiological and molecular responses of ginger to salt stress during the seedling stage. For future research directions, experimental designs to assist in mitigation can be carried out in terms of both altered inter-root environment and foliar spraying.

## Data availability statement

The original contributions presented in the study are publicly available. This data can be found here: NCBI, PRJNA898683.

## Author contributions

ML and KX conceived the experiments. ML and BC performed the experiments. ML and ZC analyzed the data. ML, YL, ZC, and KX wrote and revised the manuscript. All authors contributed to the article and approved the submitted version.
