# Supplementary material for: Physiological and molecular mechanism of ginger (Zingiber officinale Roscoe) seedling response to salt stress
Source: Front Plant Sci. 2023 Mar 17;14:1073434. doi: 10.3389/fpls.2023.1073434 (PMC10064006; doi:10.3389/fpls.2023.1073434)
Supplement: Supplementary file 1 [file Table_1.pdf]

Gene ID	Primer name	Primer sequence
121992914	121992914-F	GAAGAGGAGGAGGAGGACGAAGG
	121992914-R	GAACTTCCGCCACGATGTCCAG
121982991	121982991-F	AGTACCTGGACATGGCGGAAGG
	121982991-R	CGTCGTCGTAGTCCTCATCATTGG
122051202	122051202-F	GAGGACCGACAACGAGATCAAGAAC
	122051202-R	TTCCTCCTTAAACGCTTCGCTGTC
122053500	122053500-F	CAATGGCTGCGGAGGGAGAAAG
	122053500-R	GCTTCCACCTTCAGACGAACCAG
122016900	122016900-F	CGACAACGACGAGGAGAAACAGG
	122016900-R	CGCCCAGAGACAGTCCAATGATTAC
121974527	121974527-F	ATTTCCCTCCTCCATCCCTGTCTG
	121974527-R	TCTGGTGTGTGCTAACTGGTTGC
122033551	122033551-F	CGACGACCAGGTGCTCAAGAAG
	122033551-R	GTGAGGTTCTGTTGGGCGTATGG
122028114	122028114-F	AGTTGCCATCCGAATCCCAAAGAG
	122028114-R	ACCGTATCCGACAGGCTCATCC
121985721	121985721-F	CGCCACCCTCCTCCTCTTACTC
	121985721-R	CTCCACGACGGACAGAACGATG
